# Mitoepigenetics and Neurodegenerative Diseases

**DOI:** 10.3389/fendo.2019.00086

**Published:** 2019-02-19

**Authors:** Fabio Coppedè, Andrea Stoccoro

**Affiliations:** Medical Genetics Laboratory, Department of Translational Research and New Technologies in Medicine and Surgery, University of Pisa, Pisa, Italy

**Keywords:** mitochondrial DNA methylation, mitoepigenetics, D-loop methylation, Alzheimer's disease, Parkinson's disease, amyotrophic lateral sclerosis, neurodegeneration

## Abstract

Mitochondrial impairment and increased oxidative stress are common features in neurodegenerative disorders, leading researchers to speculate that epigenetic changes in the mitochondrial DNA (mitoepigenetics) could contribute to neurodegeneration. The few studies performed so far to address this issue revealed impaired methylation levels of the mitochondrial regulatory region (D-loop region) in both animal models, postmortem brain regions, or circulating blood cells of patients with Alzheimer's disease, Parkinson's disease, and amyotrophic lateral sclerosis. Those studies also revealed that mtDNA D-loop methylation levels are subjected to a dynamic regulation within the progression of the neurodegenerative process, could be affected by certain neurodegenerative disease-causative mutations, and are inversely correlated with the mtDNA copy number. The methylation levels of other mtDNA regions than the D-loop have been scarcely investigated in human specimens from patients with neurodegenerative disorders or in animal models of the disease, and evidence of impaired methylation levels is often limited to a single study, making it difficult to clarify their correlation with mitochondrial dynamics and gene expression levels in these disorders. Overall, the preliminary results of the studies performed so far are encouraging making mitoepigenetics a timely and attractive field of investigation, but additional research is warranted to clarify the connections among epigenetic changes occurring in the mitochondrial genome, mitochondrial DNA dynamics and gene expression, and the neurodegenerative process.

## Introduction

**Figure 1:**
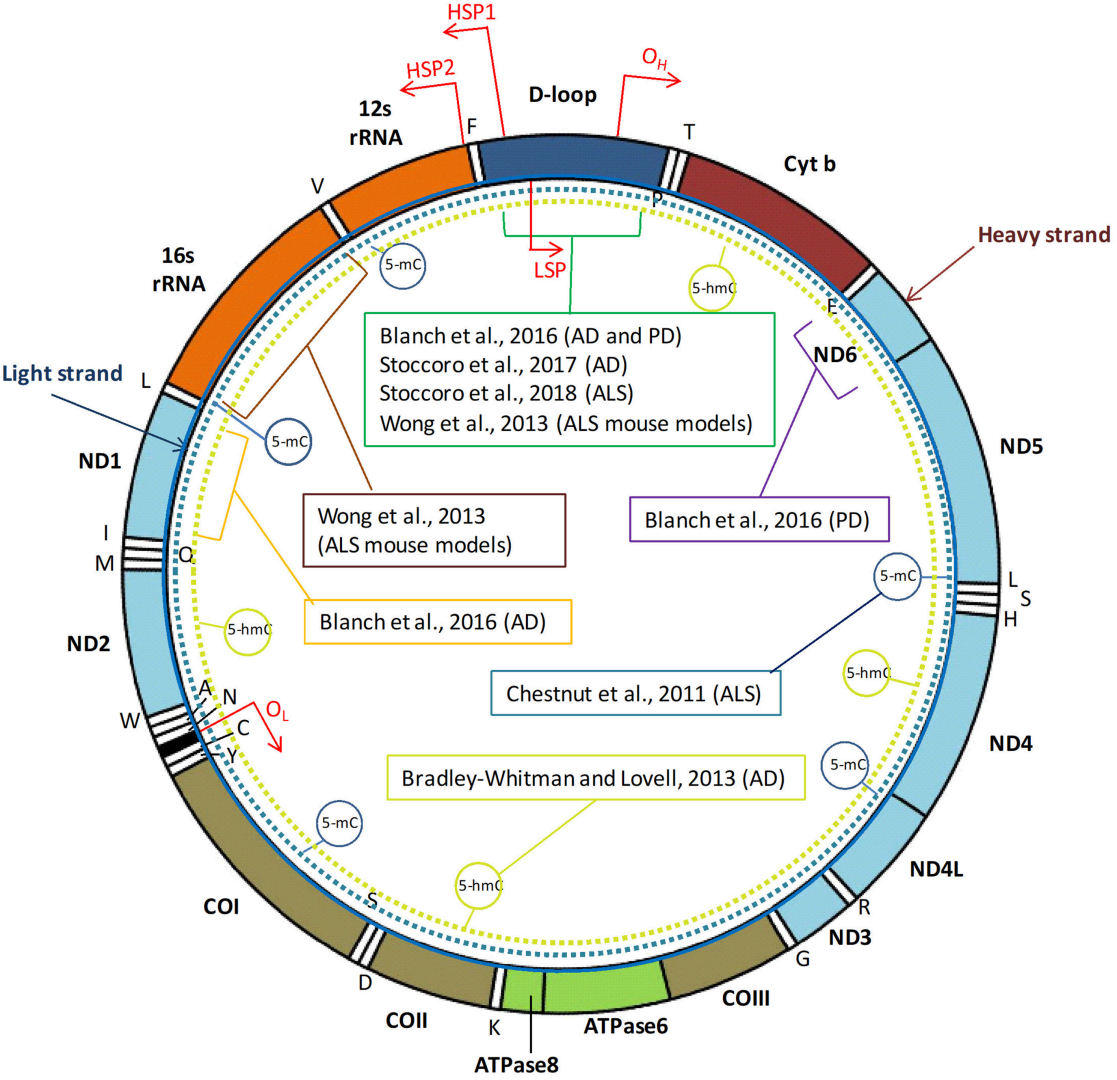
Human mtDNA organization and regions investigated by DNA methylation studies in neurodegenerative diseases. The human mitochondrial DNA (mtDNA) genome comprises 16,569 base pairs, organized as a circular double-stranded DNA formed by an inner “light” (L) strand and an outer “heavy” (H) strand. MtDNA replication initiates within the D-loop region and proceeds from the origin of heavy-strand replication (O_H_) until the origin of light-strand replication (O_L_). Three transcription promoters are present in human mtDNA, including the heavy strand promoter 1 (HSP1) that enables transcription of the two ribosomal RNAs, the HSP2 that promotes transcription of the rest of the heavy strand, and the LSP, which promotes transcription of the light strand. Positions of the two rRNA, 22 tRNA, and 13 structural genes encoded by mtDNA are indicated. MtDNA region investigated by DNA methylation analyses in tissues from patients with neurodegenerative diseases, or in animal models, are indicated with brackets. Dotted circular lines indicate studies addressing global mtDNA methylation (5-mC) or hydroxymethylation (5-hmC) levels.

Mitochondria are small cytosolic organelles evolved from a symbiotic relation between aerobic bacteria and the primordial eukaryotic cells unable to use oxygen, developed about 1.5 billion years ago. The relation became permanent as the bacteria evolved into organelles providing to the host cells the aerobic metabolism, a much more efficient way to produce energy than anaerobic glycolysis. Indeed, mitochondria have adapted to their new intracellular environment by reducing their genome size, thus increasing their replication rate and ensuring the transmission of the mitochondrial genome to two daughter cells (1). The human mitochondrial DNA (mtDNA) is a 16,569-kb circular, double-stranded molecule (Figure 1), which is present in 10^3^-10^4^ copies per cell and contains 37 genes: two rRNA genes, 22 tRNA genes, and 13 structural genes encoding subunits of the mitochondrial respiratory chain, the power station for oxidative phosphorylation (OXPHOS) and ATP synthesis (2). Particularly, mtDNA genes encode for subunits within complex I (ND1, ND2, ND3, ND4, ND4L, ND5, ND6), complex III (Cytochrome B), complex IV (COXI, COXII, COXIII), and complex V (ATPase6 and ATPase8) (3). In addition to its mRNA, rRNA, and tRNA genes, the mtDNA encompasses a non-coding region, the displacement (D) loop region, which is 1,124-bp in length, acts as a promoter for both the heavy and light strands of the mtDNA, and is critically important in the regulation of mtDNA replication and transcription (4).

Unlike the nuclear DNA, the mtDNA lacks histone-like packaging proteins and is more susceptible to oxidative damage and prone to higher rate of mutation than the nuclear genome. Neurons contain a high number of mitochondria, depend on these organelles for energy production, and are particularly vulnerable to the accumulation of mtDNA mutations with aging, that can expand to heteroplasmic levels causing respiratory chain dysfunction (5). Indeed, single nucleotide polymorphisms, deletions, insertions, and copy number variations of the mtDNA have been linked to the occurrence of neurological disorders, including neurodegenerative ones (6, 7).

Epigenetic mechanisms are a group of intracellular pathways evolved to tightly regulate gene expression levels, without changing the primary DNA sequence, and accumulating evidence supports a contribution of epigenetic modifications to the onset and progression of neurodegeneration (8–10). Increasing evidence suggests that changes in DNA methylation and hydroxymethylation occur in the mtDNA, and represent epigenetic modifications of the mitochondrial genome (mitoepigenetics) likely regulating both mtDNA replication and gene expression levels (11). Similarly, it has been largely speculated that mitoepigenetic changes could contribute to neurodegeneration (12). The present article aims to critically discuss the recent evidence of impaired mitoepigenetics in neurodegenerative disorders.

## Mitochondrial DNA Methylation

Mitoepigenetics have been neglected or denied for many years, mainly due to methodological limitations, and its occurrence is still under debate. Moreover, from an epigenetic point of view the mtDNA is largely different from the nuclear DNA. In fact mtDNA does not contain CpG islands, and it is organized into tightly packed nucleoprotein complexes called nucleoids that lack histones. Furthermore, although both long and short non-coding RNAs have been detected in human mitochondria, most of them are nuclear-encoded and later transferred into mitochondria, and until now only few non-coding RNAs, including lnc*ND5*, lnc*ND6*, and lnc*Cyt b*, have been identified to be encoded by the mtDNA itself (13). Therefore, the main epigenetic changes investigated in mtDNA are DNA methylation and hydroxymethylation (14). The presence of mtDNA methylation has been a controversial issue since the first investigations performed more than 40 years ago (15–17). Subsequent studies have continued to give conflicting results, showing either presence of mtDNA methylation in both human and mouse fibroblasts (18, 19) or no evidence of mtDNA methylation in gastric and colorectal cancer specimens (20). In 2011, a paper by Shock and collaborators reinforced the evidence of epigenetic marks in the mtDNA because the authors observed both methylated (5-mC) and hydroxymethylated (5-hmC) cytosine residues in mtDNA obtained from either human or mouse cell lines, along with the presence of DNA methyltransferase 1 (DNMT1) in mitochondria, the maintenance enzyme that transfers the methyl groups to cytosines (21).

Afterwards, other investigators detected both 5-mC and 5-hmC in mtDNA, and other DNMTs than DNMT1 have been identified in mitochondria, particularly the *de novo* DNMT3A and DNMT3B proteins (22–24). The rapid development of novel techniques for the study of nuclear DNA methylation, adapted for the study of mitochondrial epigenetic mechanisms, made possible to apply several different methods to investigate mtDNA methylation and hydroxymethylaytion. Some of such methods require the purification of mitochondria (e.g., mass spectrometry and ELISA approaches) and then detection of epigenetic marks is carried out by using antibodies against 5-mC or 5-hmC. However, bisulfite sequencing and pyrosequencing are the most widely used techniques for locus specific mtDNA measurements. Indeed, sodium bisulfite treatment converts unmethylated cytosines into uracil, and leaves 5-mC residues unaffected, thus introducing specific changes in the DNA sequence to provide single-nucleotide resolution information about its methylation status (11).

Although these methods are well established in the study of nuclear DNA methylation, some authors have raised the issue that they need to be carefully suited for the study of mtDNA methylation (25, 26). For example, it has been suggested that the mtDNA must be linearized (digested with a restriction enzyme) before bisulfite conversion, because its circular structure could affect the bisulfite conversion efficiency (25). By applying this linearization procedure several authors have suggested that mtDNA methylation is a very rare event, or even absent (25–29). However, those studies have also revealed that the detection of mtDNA methylation levels following bisulfite treatment is not only affected by the circular structure, but also by the procedures used to extract DNA samples, and by the final amount of mtDNA undergoing bisulfite treatment (25). More recently, the application of mass spectrometry, that does not require bisulfite pre-treatment for the analysis of mtDNA methylation, detected 5-mC at the levels of 0.3–0.5% of total mtDNA cytosine residues, which is equivalent to eighteen to thirty 5-mC residues per molecule of mtDNA (26). In addition, there is consensus from studies that used either linearized or circular mtDNA, as well as from studies that did not perform bisulfite treatment, that the regulatory D-loop region represents one of the most methylated sites in the mtDNA, with average methylation levels of less or about 5% (25, 30).

A deeper and clearer understanding of mitoepigenetic mechanisms could be provided by the different technologies available to assess genome-wide changes in nuclear DNA. However, as recently reviewed by Devall et al. (31), the Illumina arrays used for epigenome-wide investigations do not cover the mtDNA sequence. The methylated DNA immunoprecipitation sequencing (MeDIP-seq) approach, based on the affinity capture of methylated DNA with 5-mC antibodies, has been successfully applied to detect mtDNA methylation levels across regions of the human brain and in blood cells (30), and whole-genome bisulfite sequencing has been recently applied to study mtDNA methylation in adenomas (32). However, the higher amount of input DNA required, the relatively high costs of those techniques, and problems related to cell-type isolation, have limited the use of these approaches in favor of candidate-gene investigations. Other next generation sequencing technologies, including those related to single-molecule real-time sequencing and third generation sequencing approaches, have the potential to be implemented in the future for the study of whole mtDNA methylation and hydroxymethylation profiles (31).

Overall, it is nowadays clear that mtDNA methylation and hydroxymethylation levels are much lower than those of nuclear DNA. However, many studies indicate that although low, such methylation levels may play a role in mtDNA replication and transcription. A correlation between the mtDNA D-loop region methylation and mitochondrial copy number has been observed in human and mouse cell cultures (33, 34), in peripheral blood cells (35–39), in colorectal cancer tissues (40) and in the human placenta (41). Moreover, correlation between mtDNA methylation and gene expression has been reported in mesenchymal stem cells (42), in colorectal cancer tissues (40, 43), and in peripheral blood samples (36). Furthermore, mtDNA methylation and hydroxymethylation patterns have been linked to the exposure to various environmental agents. For instance, it has been reported that mtDNA methylation is modulated by exposure to particulate matter (PM_1_ and PM_2.5_), air benzene, traffic-derived elemental carbon (35, 41, 44), particle-containing welding fumes (38), endocrine disruptors (45), maternal smoking (46), chrome (47), arsenic (36) as well as to the pharmacological agent valproic acid (48). Variations in mtDNA methylation patterns have been also associated to various endogenous metabolites, including thyroid hormones (49), homocysteine (50) and glucose (51, 52). Also cell senescence (33, 42) and aging (53–55) modulate mtDNA methylation levels. Moreover, mtDNA methylation patterns have been associated to various human diseases, including cardiovascular diseases (56), colorectal cancer (40, 43, 57), nonalcoholic steathopatitis (58), obesity (39), and Down's syndrome (59), as well as to different neurodegenerative diseases.

## Mitoepigenetics And Neurodegenerative Diseases

Table 1 summarizes the studies performed so far with the aim to investigate mtDNA methylation and hydroxymethylation in animal models of neurodegeneration or in human samples from patients suffering from these disorders, and Figure 1 shows the mitochondrial regions and/or the genes investigated in those studies. Early in 2011, Chestnut and coworkers investigated the global 5-mC content and DNMT protein levels in nuclei and mitochondria from both brain and spinal cord motor neurons of mice, as well as in cortical motor neurons from 12 patients with amyotrophic lateral sclerosis (ALS), revealing that motor neurons engage epigenetic mechanisms to drive apoptosis, involving up-regulation of DNMTs and increased global DNA methylation in both nuclei and mitochondria (22). Subsequently, the same group revealed that mtDNA methylation patterns and mitochondrial DNMT3A levels are abnormal in skeletal muscles and spinal cord of pre-symptomatic ALS mice that carry mutations in the human superoxide dismutase 1 gene (*SOD1*), including DNMT3A up-regulation, increased 16S rRNA gene methylation, and decreased D-loop region methylation (24). More recently, we investigated blood DNA samples from 114 individuals, including ALS patients, presymptomatic carriers of ALS-causative mutations, and noncarrier family members, and observed a significantly decreased methylation of the mtDNA D-loop region in carriers of ALS-linked *SOD1* mutations (either ALS patients or presymtomatic carriers) with respect to noncarriers of ALS-gene mutations or to carriers of mutations in other ALS-causative genes (*FUS, TARDBP, C9orf72*). Moreover, we observed a strong inverse correlation between D-loop methylation levels and the mtDNA copy number in our cohort and, due to the fact that SOD1 is one of the major antioxidant enzymes, we hypothesized that the observed D-loop hypomethylation in *SOD1* carriers could represent a mechanism to increase mtDNA replication to counteract the increased burden of oxidative damage (37). Collectively, these studies suggest that mtDNA methylation is impaired in ALS tissues, and could represent an early event preceding the onset of disease symptoms in carriers of *SOD1* mutations.

**Table 1:** MtDNA methylation investigations in neurodegenerative diseases.

**Experimental model**	**Method of measurement**	**mtDNA region investigated**	**Observation**	**References**
Brain and spinal cord motor neurons of mice, and post-mortem human cortex of 12 ALS patients	Immunohistochemistry	Global 5-mC levels	Increased DNMT activity and 5-mC levels in motor neurons.	(22)
Spinal cord and skeletal muscle of ALS mice and non transgenic (non-tg) mice	Pyrosequencing	D-loop and 16S rRNA	Higher methylation levels of 16S rRNA gene in spinal cord and skeletal muscle of mice with G37R and G93A *SOD1* mutations, and lower methylation in *SOD1* wild-type mice respect to non-tg mice. Decreased D-loop methylation in spinal cord of mice with G93A *SOD1* mutation respect to non-tg mice.	(24)
Superior and middle temporal gyrus (SMTG) and cerebellum (CER) of 7 late-onset AD patients and 5 control subjects	Immunohistochemistry	Global 5-hmC levels	A trend toward a significant increase in SMTG mtDNA of AD patients compared to control subjects.	(60)
Entorhinal cortex of 8 AD-related pathology patients and 8 control subjects, cerebral cortex of AD mouse model, and *substantia nigra* of 10 PD patients and 10 control subjects	Pyrosequencing	Methylation and hydroxymethylation levels of D-loop region, *MT-ND1*, and *MT-ND6* genes	Increased D-loop methylation levels in AD related pathology patients respect to control subjects. Dynamic pattern of D-loop methylation in mice along with Alzheimer disease pathology progression. Decreased D-loop methylation found in PD patients respect to control subjects. *MT-ND1* less methylated in AD-related patients than in control samples.	(61)
Peripheral blood of 133 late-onset AD patients and 130 matched controls	Methylation sensitive-high resolution melting	D-loop region	Significant reduction of D-loop methylation in AD patients.	(62)
Peripheral blood of 114 individuals, including 54 ALS patients, 28 presymptomatic carriers, and 32 noncarrier family members	Methylation sensitive-high resolution melting	D-loop region	*SOD1* mutation carriers showed a significant decrease in D-loop methylation levels. Inverse correlation between D-loop methylation levels and mtDNA copy number.	(37)

A not significant increase of 5-hmC levels was observed in post-mortem mtDNA brain samples (temporal gyrus) of seven late-onset Alzheimer's disease (AD) patients with respect to five matched controls (60). A more recent study revealed increased methylation levels of the mtDNA D-loop region in the entorhinal cortex of 8 patients with AD-related pathology (stages I/II and III/IV of Braak), but no significant difference in the content of mitochondrial 5-hmC levels was observed between AD patients and matched controls. The degree of D-loop region methylation was higher in early disease stages than in later stages, and these results were corroborated by a dynamic pattern of methylation of this region that was observed in transgenic AD mice (APP/PS1 mice) along with disease progression (61). We next assessed the methylation levels of the mtDNA D-loop region in blood DNA samples from 133 late-onset AD patients and 130 matched controls, observing a significant reduced methylation in the first group (62). Decreased D-loop methylation levels were also observed post-mortem in the *substantia nigra* of ten patients with Parkinson's disease (PD) respect to healthy matched controls (61). Collectively, these data suggest that the degree of methylation of this region might characterize different stages of the neurodegenerative process.

The methylation levels of other mtDNA regions than the D-loop have been scarcely investigated in human specimens from patients with neurodegenerative disorders, and data are available only for *MT*-*ND1* and *MT*-*ND6* genes (61). Particularly, reduced *MT-ND1* methylation levels were observed in the enthorinal cortex of 8 patients with AD-related pathology with respect to healthy control brains, and no difference in *MT*-*ND6* methylation levels was observed between post-mortem PD and control brains. No changes in *MT*-*ND1* and *MT*-*ND6* hydroxymethylation levels were observed in post-mortem AD or PD brain regions, respectively (61).

## Conclusions and Future Perspectives

Overall, only a few studies have so far investigated mitoepigenetic changes in human specimens or animal models of neurodegenerative disorders, providing encouraging but still preliminary results (Table 1). Most of these studies investigated the methylation levels of the mitochondrial D-loop region (24, 37, 61, 62), and there is consensus in the literature that the methylation levels of this region correlate with the copy number of the mtDNA. Particularly, an inverse correlation between D-loop methylation levels and mtDNA copy number was observed in cancerous tissues (40), in human placenta (41), and in peripheral blood DNA samples (35, 36, 38, 39), including blood DNA samples of patients with neurodegenerative diseases (37). Furthermore, it has been suggested that the D-loop methylation status is subjected to dynamic changes along with the progression of the neurodegenerative process in brain regions (61). Our recent investigation in carriers of different ALS-linked mutations, and particularly in carriers of mutations in *SOD1, FUS, TARDBP*, and *C9orf72*, that represent the major ALS causative genes, revealed that only carriers of *SOD1* mutations showed a significant hypomethylation of the mtDNA D-loop region, suggesting that it could represent a compensatory mechanism to oxidative stress, leading to an increased mtDNA copy number (37). Therefore, despite that there is substantial evidence of impaired mtDNA D-loop region methylation in both humans and animal models of neurodegeneration, further studies are required to clarify the biological significance of these epigenetic changes and their cause/consequence relationship with the neurodegenerative process.

Concerning the coding regions of the mitochondrial DNA, the investigation of their methylation status in patients with neurodegenerative diseases is limited to a few subjects (61), making it difficult to clarify the relationship between their methylation levels and the resulting gene expression levels. In this regard, recent studies investigating the expression levels of mitochondrial genes in early and late stages of the neurodegenerative process provided conflicting results (63, 64), and the question is still open. In addition to studies directly investigating mtDNA methylation in patients with neurodegenerative disorders, it has been also recently suggested that mitoepigenetic changes could result from traumatic brain injury, a well-known risk factor for neurodegeneration (65). However, despite that there is evidence suggesting that different mtDNA haplogroups and changes in nuclear DNA methylation play a role in the onset of traumatic brain injury, a direct involvement of mitoepigenetics is only supposed and deserves further investigation (65). Overall, the field of mitoepigenetics in neurodegenerative diseases is a timely and attractive area of investigation, preliminary results are encouraging, but additional research is warranted to clarify the connections among epigenetic changes occurring in the mitochondrial genome, mitochondrial DNA dynamics and gene expression, and the neurodegenerative process.

## Author Contributions

FC and AS performed literature search, drafted the manuscript, and contributed to the discussion.

### Conflict of Interest Statement

The authors declare that the research was conducted in the absence of any commercial or financial relationships that could be construed as a potential conflict of interest.

## References

[1] DiMauro S Schon EA. Mitochondrial respiratory-chain diseases: mitochondrial respiratory-chain diseases. N Engl J Med. (2003) 348:2656–68. 10.1056/NEJMra022567 12826641

[2] Taanman JW The mitochondrial genome: structure, transcription, translation and replication. Biochim Biophys Acta (1999) 9:103–23. 10.1016/S0005-2728(98)00161-3 10076021

[3] Tyagi A Pramanik R Vishnubhatla S Ali S Bakhshi R Chopra A. Pattern of mitochondrial D-loop variations and their relation with mitochondrial encoded genes in pediatric acute myeloid leukemia. Mutat Res. (2018) 810:13–8. 10.1016/j.mrfmmm.2018.05.002 29883862

[4] Sbisà E Tanzariello F Reyes A Pesole G Saccone C. Mammalian mitochondrial D-loop region structural analysis: identification of new conserved sequences and their functional and evolutionary implications. Gene (1997) 205:125–40. 9461386 10.1016/s0378-1119(97)00404-6

[5] Keogh MJ Chinnery PF. Mitochondrial DNA mutations in neurodegeneration. Biochim Biophys Acta (2015) 1847:1401–11. 10.1016/j.bbabio.2015.05.015 26014345

[6] Coppedè F Migliore L. DNA damage in neurodegenerative diseases. Mutat Res. (2015) 776:84–97. 10.1016/j.mrfmmm.2014.11.010 26255941

[7] Cruz ACP Ferrasa A Muotri AR Herai RH Frequency and association of mitochondrial genetic variants with neurological disorders. Mitochondrion (2018) S1567–7249(18)30113–2. 10.1016/j.mito.2018.09.005. [Epub ahead of print]. 30218715

[8] Berson A Nativio R Berger SL Bonini NM. Epigenetic regulation in neurodegenerative diseases. Trends Neurosci. (2018) 41:587–98. 10.1016/j.tins.2018.05.005 29885742 PMC6174532

[9] Gangisetty O Cabrera MA Murugan S. Impact of epigenetics in aging and age related neurodegenerative diseases. Front Biosci. (2018) 23:1445–64 10.2741/4654 29293444

[10] Stoccoro A Coppedè F. Role of epigenetics in Alzheimer's disease pathogenesis. Neurodegener Dis Manag. (2018) 8:181–93. 10.2217/nmt-2018-0004 29888987

[11] van der Wijst MG Rots MG. Mitochondrial epigenetics: an overlooked layer of regulation? Trends Genet. (2015) 31:353–6. 10.1016/j.tig.2015.03.009 25891224

[12] Devall M Mill J Lunnon K. The mitochondrial epigenome: a role in Alzheimer's disease? Epigenomics (2014) 6:665–75. 10.2217/epi.14.50 25531259 PMC4329914

[13] Rackham O Shearwood AM Mercer TR Davies SM Mattick JS Filipovska A. Long noncoding RNAs are generated from the mitochondrial genome and regulated by nuclear-encoded proteins. RNA (2011) 17:2085–93. 10.1261/rna.029405.111 22028365 PMC3222122

[14] Stimpfel M Jancar N Virant-Klun I. New challenge: mitochondrial epigenetics? Stem Cell Rev. (2018) 14:13–26. 10.1007/s12015-017-9771-z 28980199

[15] Vanyushin BF Kiryanov GI Kudryashova IB Belozersky AN. DNA-methylase in loach embryos (Misgurnus fossilis). FEBS Lett. (1971) 15:313–6. 10.1016/0014-5793(71)80646-4 11945872

[16] Nass MM. Differential methylation of mitochondrial and nuclear DNA in cultured mouse, hamster and virus-transformed hamster cells. *In vivo* and *in vitro* methylation. J Mol Biol. (1973) 80:155–75. 10.1016/0022-2836(73)90239-8 4361747

[17] Cummings DJ Tait A Goddard JM. Methylated bases in DNA from *Paramecium aurelia*. Biochim Biophys Acta (1974) 374:1–11. 10.1016/0005-2787(74)90194-4 4429738

[18] Shmookler Reis RJ Goldstein S. Mitochondrial DNA in mortal and immortal human cells. Genome number, integrity, and methylation. J Biol Chem. (1983) 258:9078–85. 6307991

[19] Pollack Y Kasir J Shemer R Metzger S Szyf M. Methylation pattern of mouse mitochondrial DNA. Nucleic Acids Res. (1984) 12:4811–24. 10.1093/nar/12.12.4811 6330684 PMC318881

[20] Maekawa M Taniguchi T Higashi H Sugimura H Sugano K Kanno T Methylation of mitochondrial DNA is not a useful marker for cancer detection. Clin Chem. (2004) 50:1480–1. 10.1373/clinchem.2004.035139 15277367

[21] Shock LS Thakkar PV Peterson EJ Moran RG Taylor SM. DNA methyltransferase 1, cytosine methylation, and cytosine hydroxymethylation in mammalian mitochondria. Proc Natl Acad Sci USA. (2011) 108:3630–5. 10.1073/pnas.1012311108 21321201 PMC3048134

[22] Chestnut BA Chang Q Price A Lesuisse C Wong M Martin LJ. Epigenetic regulation of motor neuron cell death through DNA methylation. J Neurosci. (2011) 31:16619–36. 10.1523/JNEUROSCI.1639-11.2011 22090490 PMC3238138

[23] Bellizzi D D'Aquila P Scafone T Giordano M Riso V Riccio A. The control region of mitochondrial DNA shows an unusual CpG and non-CpG methylation pattern. DNA Res. (2013) 20:537–47. 10.1093/dnares/dst029 23804556 PMC3859322

[24] Wong M Gertz B Chestnut BA Martin LJ. Mitochondrial DNMT3A and DNA methylation in skeletal muscle and CNS of transgenic mouse models of ALS. Front Cell Neurosci. (2013) 7:279. 10.3389/fncel.2013.00279 24399935 PMC3872319

[25] Liu B Du Q Chen L Fu G Li S Fu L. CpG methylation patterns of human mitochondrial DNA. Sci Rep. (2016) 6:23421. 10.1038/srep23421 26996456 PMC4800444

[26] Matsuda S Yasukawa T Sakaguchi Y Ichiyanagi K Unoki M Gotoh K. Accurate estimation of 5-methylcytosine in mammalian mitochondrial DNA. Sci Rep. (2018) 8:5801. 10.1038/s41598-018-24251-z 29643477 PMC5895755

[27] Hong EE Okitsu CY Smith AD Hsieh CL. Regionally specific and genome-wide analyses conclusively demonstrate the absence of CpG methylation in human mitochondrial DNA. Mol Cell Biol. (2013) 33:2683–90. 10.1128/MCB.00220-13 23671186 PMC3700126

[28] Mechta M Ingerslev LR Fabre O Picard M Barrès R. Evidence suggesting absence of mitochondrial DNA methylation. Front Genet. (2017) 8:166. 10.3389/fgene.2017.00166 29163634 PMC5671948

[29] Owa C Poulin M Yan L Shioda T. Technical adequacy of bisulfite sequencing and pyrosequencing for detection of mitochondrial DNA methylation: sources and avoidance of false-positive detection. PLoS ONE (2018) 13:e0192722. 10.1371/journal.pone.0192722 29420656 PMC5805350

[30] Devall M Smith RG Jeffries A Hannon E Davies MN Schalkwyk L. Regional differences in mitochondrial DNA methylation in human post-mortem brain tissue. Clin Epigenetics (2017) 9:47. 10.1186/s13148-017-0337-3 28473874 PMC5415779

[31] Devall M Roubroeks J Mill J Weedon M Lunnon K. Epigenetic regulation of mitochondrial function in neurodegenerative disease: new insights from advances in genomic technologies. Neurosci Lett. (2016) 625:47–55. 10.1016/j.neulet.2016.02.013 26876477 PMC5747527

[32] Morris MJ Hesson LB Poulos RC Ward RL Wong JWH Youngson NA Reduced nuclear DNA methylation and mitochondrial transcript changes in adenomas do not associate with mtDNA methylation. Biomark Res. (2018) 6:37 10.1186/s40364-018-0151-x 30619609 PMC6311003

[33] Bianchessi V Vinci MC Nigro P Rizzi V Farina F Capogrossi MC. Methylation profiling by bisulfite sequencing analysis of the mtDNA Non-Coding Region in replicative and senescent endothelial cells. Mitochondrion (2016) 27:40–7. 10.1016/j.mito.2016.02.004 26910457

[34] van der Wijst MG van Tilburg AY Ruiters MH Rots MG Experimental mitochondria-targeted DNA methylation identifies GpC methylation, not CpG methylation, as potential regulator of mitochondrial gene expression. Sci Rep. (2017) 7:177 10.1038/s41598-017-00263-z 28282966 PMC5428053

[35] Byun HM Panni T Motta V Hou L Nordio F Apostoli P. Effects of airborne pollutants on mitochondrial DNA methylation. Part Fibre Toxicol. (2013) 10:18. 10.1186/1743-8977-10-18 23656717 PMC3660297

[36] Sanyal T Bhattacharjee P Bhattacharjee S Bhattacharjee P. Hypomethylation of mitochondrial D-loop and ND6 with increased mitochondrial DNA copy number in the arsenic-exposed population. Toxicology (2018) 408:54–61. 10.1016/j.tox.2018.06.012 29940200

[37] Stoccoro A Mosca L Carnicelli V Cavallari U Lunetta C Marocchi A. Mitochondrial DNA copy number and D-loop region methylation in carriers of amyotrophic lateral sclerosis gene mutations. Epigenomics (2018) 10:1431–43. 10.2217/epi-2018-0072 30088417

[38] Xu Y Li H Hedmer M Hossain MB Tinnerberg H Broberg K. Occupational exposure to particles and mitochondrial DNA—relevance for blood pressure. Environ Health (2017) 16:22. 10.1186/s12940-017-0234-4 28274239 PMC5343309

[39] Zheng LD Linarelli LE Liu L Wall SS Greenawald MH Seidel RW. Insulin resistance is associated with epigenetic and genetic regulation of mitochondrial DNA in obese humans. Clin Epigenetics (2015) 7:60. 10.1186/s13148-015-0093-1 26110043 PMC4479353

[40] Gao J Wen S Zhou H Feng S. De-methylation of displacement loop of mitochondrial DNA is associated with increased mitochondrial copy number and nicotinamide adenine dinucleotide subunit 2 expression in colorectal cancer. Mol Med Rep. (2015) 12:7033–8. 10.3892/mmr.2015.4256 26323487

[41] Janssen BG Byun HM Gyselaers W Lefebvre W Baccarelli AA Nawrot TS. Placental mitochondrial methylation and exposure to airborne particulate matter in the early life environment: an ENVIRONAGE birth cohort study. Epigenetics (2015) 10:536–44. 10.1080/15592294.2015.1048412 25996590 PMC4623402

[42] Yu D Du Z Pian L Li T Wen X Li W. Mitochondrial DNA hypomethylation is a biomarker associated with induced senescence in human fetal heart mesenchymal stem cells. Stem Cells Int. (2017) 2017:1764549. 10.1155/2017/1764549 28484495 PMC5397648

[43] Feng S Xiong L Ji Z Cheng W Yang H. Correlation between increased ND2 expression and demethylated displacement loop of mtDNA in colorectal cancer. Mol Med Rep. (2012) 6:125–30. 10.3892/mmr.2012.870 22505229

[44] Byun HM Colicino E Trevisi L Fan T Christiani DC Baccarelli AA. Effects of air pollution and blood mitochondrial DNA methylation on markers of heart rate variability. J Am Heart Assoc. (2016) 5:e003218. 10.1161/JAHA.116.003218 27107129 PMC4843532

[45] Byun HM Benachour N Zalko D Frisardi MC Colicino E Takser L. Epigenetic effects of low perinatal doses of flame retardant BDE-47 on mitochondrial and nuclear genes in rat offspring. Toxicology (2015) 328:152–9. 10.1016/j.tox.2014.12.019 25533936 PMC4353575

[46] Armstrong DA Green BB Blair BA Guerin DJ Litzky JF Chavan NR. Maternal smoking during pregnancy is associated with mitochondrial DNA methylation. Environ Epigenet. (2016) 2:dvw020. 10.1093/eep/dvw020 28979800 PMC5624552

[47] Yang L Xia B Yang X Ding H Wu D Zhang H. Mitochondrial DNA hypomethylation in chrome plating workers. Toxicol Lett. (2016) 243:1–6. 10.1016/j.toxlet.2015.11.031 26656300

[48] Chen H Dzitoyeva S Manev H. Effect of valproic acid on mitochondrial epigenetics. Eur J Pharmacol. (2012) 690:51–9. 10.1016/j.ejphar.2012.06.019 22728245 PMC3419440

[49] Janssen BG Byun HM Roels HA Gyselaers W Penders J Baccarelli AA. Regulating role of fetal thyroid hormones on placental mitochondrial DNA methylation: epidemiological evidence from the ENVIRONAGE birth cohort study. Clin Epigenetics (2017) 9:66. 10.1186/s13148-017-0366-y 28649287 PMC5479026

[50] Jia L Li J He B Jia Y Niu Y Wang C. Abnormally activated one-carbon metabolic pathway is associated with mtDNA hypermethylation and mitochondrial malfunction in the oocytes of polycystic gilt ovaries. Sci Rep. (2016) 6:19436. 10.1038/srep19436 26758245 PMC4725837

[51] Mishra M Kowluru RA. Epigenetic modification of mitochondrial DNA in the development of diabetic retinopathy. Invest Ophthalmol Vis Sci. (2015) 56:5133–42. 10.1167/iovs.15-16937 26241401 PMC4525675

[52] Zheng LD Linarelli LE Brooke J Smith C Wall SS Greenawald MH. Mitochondrial epigenetic changes link to increased diabetes risk and early-stage prediabetes indicator. Oxid Med Cell Longev. (2016) 2016:5290638. 10.1155/2016/5290638 27298712 PMC4889851

[53] Dzitoyeva S Chen H Manev H. Effect of aging on 5-hydroxymethylcytosine in brain mitochondria. Neurobiol Aging (2012) 33:2881–91. 10.1016/j.neurobiolaging.2012.02.006 22445327 PMC3462297

[54] Mawlood SK Dennany L Watson N Dempster J Pickard BS. Quantification of global mitochondrial DNA methylation levels and inverse correlation with age at two CpG sites. Aging (2016) 8:636–41. 10.18632/aging.100892 26887692 PMC4925819

[55] D'Aquila P Giordano M Montesanto A De Rango F Passarino G Bellizzi D. Age-and gender-related pattern of methylation in the MT-RNR1 gene. Epigenomics (2015) 7:707–16. 10.2217/epi.15.30 26343273

[56] Baccarelli AA Byun HM. Platelet mitochondrial DNA methylation: a potential new marker of cardiovascular disease. Clin Epigenetics (2015) 7:44. 10.1186/s13148-015-0078-0 25901189 PMC4404685

[57] Tong H Zhang L Gao J Wen S Zhou H Feng S. Methylation of mitochondrial DNA displacement loop region regulates mitochondrial copy number in colorectal cancer. Mol Med Rep. (2017) 16:5347–53. 10.3892/mmr.2017.7264 28849075 PMC5647067

[58] Pirola CJ Gianotti TF Burgueño AL Rey-Funes M Loidl CF Mallardi P. Epigenetic modification of liver mitochondrial DNA is associated with histological severity of nonalcoholic fatty liver disease. Gut (2013) 62:1356–63. 10.1136/gutjnl-2012-302962 22879518

[59] Infantino V Castegna A Iacobazzi F Spera I Scala I Andria G. Impairment of methyl cycle affects mitochondrial methyl availability and glutathione level in Down's syndrome. Mol Genet Metab. (2011) 102:378–82. 10.1016/j.ymgme.2010.11.166 21195648

[60] Bradley-Whitman MA Lovell MA. Epigenetic changes in the progression of Alzheimer's disease. Mech Ageing Dev. (2013) 134:486–95. 10.1016/j.mad.2013.08.005 24012631 PMC3857018

[61] Blanch M Mosquera JL Ansoleaga B Ferrer I Barrachina M. Altered mitochondrial DNA methylation pattern in Alzheimer disease-related pathology and in Parkinson disease. Am J Pathol. (2016) 186:385–97. 10.1016/j.ajpath.2015.10.004 26776077

[62] Stoccoro A Siciliano G Migliore L Coppedè F. Decreased methylation of the mitochondrial D-Loop region in late-onset Alzheimer's disease. J Alzheimers Dis. (2017) 59:559–64. 10.3233/JAD-170139 28655136

[63] Lunnon K Keohane A Pidsley R Newhouse S Riddoch-Contreras J Thubron EB. Mitochondrial genes are altered in blood early in Alzheimer's disease. Neurobiol Aging (2017) 53:36–47. 10.1016/j.neurobiolaging.2016.12.029 28208064

[64] Mastroeni D Khdour OM Delvaux E Nolz J Olsen G Berchtold N Nuclear but not mitochondrial-encoded oxidative phosphorylation genes are altered in aging, mild cognitive impairment, and Alzheimer's disease. Alzheimers Dement. (2017) 13:510–9. 10.1016/j.jalz.2016.09.003 27793643 PMC5967608

[65] Wong VS Langley B. Epigenetic changes following traumatic brain injury and their implications for outcome, recovery and therapy. Neurosci Lett. (2016) 625:26–33. 10.1016/j.neulet.2016.04.009 27155457 PMC4915732

